# Evaluating the Serum Levels of Beclin-1 and Mammalian/Mechanistic Target of Rapamycin (mTOR) in Three Different Professional Categories

**DOI:** 10.7759/cureus.45335

**Published:** 2023-09-16

**Authors:** Piercarlo Minoretti, Ángel García Martín, Manuel Gómez Serrano, Andrés Santiago Sáez, Miryam Liaño Riera, Enzo Emanuele

**Affiliations:** 1 General Direction, Studio Minoretti, Oggiono, ITA; 2 Legal Medicine, Psychiatry and Pathology, Complutense University of Madrid, Madrid, ESP; 3 Legal Medicine, Hospital Clinico San Carlos, Madrid, ESP; 4 Occupational Health, 2E Science, Robbio, ITA

**Keywords:** laboratory medicine, occupational medicine, mammalian/mechanistic target of rapamycin, beclin-1, autophagy

## Abstract

Background: The possible associations between occupational factors and autophagy - a catabolic process that is evolutionarily conserved and serves as a vital cornerstone in maintaining cellular balance - remain largely unexplored.

Objectives: We assessed serum levels of beclin-1, a principal effector of autophagy, and the mammalian/mechanistic target of rapamycin (mTOR), a protein recognized for its part in suppressing autophagy, within a group of healthy individuals hailing from three different professional fields, each characterized by its unique working conditions.

Methods: A total of 60 men were recruited from three distinct occupational categories: airline pilots, construction laborers, and fitness trainers. Each group consisted of 20 subjects who were selected during routine occupational health appointments. Serum levels of beclin-1 and mTOR were measured using commercially available immunoassays and compared among the three categories.

Results: Fitness instructors had the highest concentration of beclin-1 (3.1 ± 0.9 ng/mL). Construction workers followed with a mean of 2.4 ± 0.4 ng/mL, while airline pilots had the lowest levels at 1.9 ± 0.5 ng/mL (one-way analysis of variance, P < 0.001). In terms of mTOR levels, construction workers had the highest concentration (5.9 ± 1.9 ng/mL), followed by airline pilots (4.4 ± 1.7 ng/mL). Fitness instructors, on the other hand, had the lowest mTOR levels (3.5 ± 1.2 ng/mL; one-way analysis of variance, P < 0.001).

Conclusions: Serum levels of autophagy biomarkers can vary among healthy individuals based on their professional roles. Considering the crucial function autophagy serves in both health and disease, further investigations are crucial to deepen our comprehension of the potential implications of autophagy in the field of occupational medicine.

## Introduction

Autophagy, an evolutionarily preserved catabolic process, serves as a crucial pillar in sustaining cellular homeostasis and enhancing survival by systematically degrading and recycling obsolete or superfluous macromolecules and organelles [1,2]. This cellular pathway operates at a relatively low level under physiological conditions but exhibits a high degree of inducibility in response to different forms of cellular stress [3]. Enhancing the autophagic flow can bolster resistance to stress and curtail genetic damage, making it an essential part of cellular health [4]. However, any disruption or dysregulation of autophagy can pave the way for a myriad of pathological conditions, ranging from cancer to cardiovascular diseases and various metabolic disorders [2,5]. In recent years, a multitude of studies have delved into the health risks among different occupational groups including, among others, manual laborers [6], long-haul drivers [7], healthcare providers [8], and firefighters [9]. Furthermore, correlations between occupational factors and mortality rates have been observed [10], though the underlying biological mechanisms have yet to be sufficiently scrutinized. While the critical role of autophagy in safeguarding overall health is well-recognized, its potential influence in the context of occupational medicine has remained largely unexplored.

Airline pilots face distinctive occupational circumstances that are marked by irregular schedules, prolonged periods of wakefulness, and extended exposure to ultraviolet and cosmic radiation [11,12] Such conditions are recognized contributors to an increased risk of cardiometabolic disorders and malignant melanoma [11,12], all conditions that could be closely linked to compromised autophagy flux [13-15]. Additionally, construction workers who are regularly exposed to environmental contaminants like particulate matter and toxic metals may also experience an increase in autophagic flux as a protective response [16]. Recently, Rahman et al. [16] have shed light on the complex interactions between environmental pollutants, to which construction workers are often exposed, and their potential effect on autophagic flux. They highlighted three primary interactions: amplifying the flux as a defensive response, inhibiting its activity, or transforming its protective role into a mechanism that promotes cellular death [16]. Conversely, professions that involve an active lifestyle, such as fitness instructors, are anticipated to present elevated baseline levels of exercise-induced autophagy [17]. This is turn tied to a multitude of metabolic and cardiovascular benefits inherent in consistent physical engagement [18].

Prior research has delved into the exploration of different serum biomarkers associated with autophagy in patients suffering from diverse disease conditions [2]. Among these, beclin-1 carries particular importance as it plays a direct role in the formation of autophagosomes [19]. A decrease in serum beclin-1 concentrations signifies a disruption in the autophagy flux at a systemic level, which could potentially lead to negative implications on overall health and longevity [20-22]. Conversely, the mammalian/mechanistic target of rapamycin (mTOR), a protein known for its role in hindering autophagy, has been associated with cellular aging [21]. This association has established mTOR as a dependable biomarker for cellular senescence. An increase in serum mTOR levels might serve as a sign of disrupted autophagy, potentially triggering cellular dysfunction and degenerative processes [21].

In this study, we measured the serum levels of beclin-1 and mTOR in three distinct professional categories - airline pilots, construction workers, and fitness instructors. By including a diverse range of occupational groups, we were able to analyze and compare the potential variations in autophagic responses directly linked to diverse job types. Our findings could potentially yield insights into how professional factors influence autophagy, thereby providing new avenues for assessing risks, promoting protective measures, and devising preventive strategies.

## Materials and methods

Study population

This research is an integral part of an ongoing comprehensive effort to identify the biomarker profile of various professional groups [23,24]. We recruited a convenience sample of 60 male participants from three different professional fields: airline pilots, construction workers, and fitness trainers. Each group consisted of 20 individuals. The participants were selected during regular occupational health evaluations at outpatient clinics. Due to the small sample size, we did not include women in the study. We also excluded individuals with a history of psychiatric, neurological, autoimmune, inflammatory, or infectious conditions, as well as those with malignancies or recent pharmacological treatment. Additionally, none of the participants were using any supplements and all appeared to be in good physical health. During clinical interviews, no subjects reported significant work-related stress or burnout. The study was approved by the local ethics committee (reference number: 2022/12), and all participants provided written informed consent.

Quantification of serum beclin-1 and mTOR levels

Following an overnight fast, peripheral blood samples were drawn through venipuncture into serum separator tubes and centrifuged at 3000 g for 10 min. The serum was partitioned into aliquots and preserved at a temperature of -20° for subsequent assays. Multiple freeze-thaw cycles were avoided. We quantified the serum concentrations of the autophagy biomarkers, beclin-1 and mTOR, using commercial ELISA kits (MyBioSource Inc., San Diego, CA, USA), strictly adhering to the manufacturer’s protocol. To calculate individual concentrations, we established standard curves for each biomarker and translated the mean fluorescence intensity from each well into a concentration using the linear portion of the corresponding standard curve. Each biochemical evaluation was performed twice, with the results being averaged. The intra-assay and inter-assay coefficients of variation were below 7% and 9%, respectively. To minimize the risk of differential measurement errors, laboratory personnel were not provided with any details regarding the participants’ work-related information.

Data analysis

To assess data normality, we employed the Kolmogorov-Smirnov test. Continuous variables were found to have a normal distribution and were presented as mean ± standard deviation. For evaluating continuous data between the three study groups, a one-way analysis of variance (ANOVA) was conducted followed by Tukey’s post hoc tests. Categorical variables were represented as counts and percentages and analyzed using the χ2 test. Pearson’s correlation coefficient was used to investigate the associations between biomarker levels and the general characteristics of the study participants. Statistical calculations were performed using SPSS (version 20.0; IBM, Armonk, NY, USA). Two-tailed P values < 0.05 were considered statistically significant.

## Results

Table 1 provides a comparative analysis of the general characteristics of the three study groups.

**Table 1:** General characteristics of the study participants Data are expressed as mean ± standard deviation. Abbreviations: AST, aspartate aminotransferase; ALT, alanine aminotransferase; ns, not significant.

Variable	Airline pilots (n = 20)	Construction workers (n = 20)	Fitness instructors (n = 20)	P value
Men	20	20	20	ns
Age, years	39.2 ± 3.3	38.9 ± 3.4	38.1 ± 2.1	ns
Body mass index, kg/m^2^	23.8 ± 2.3	23.9 ± 2.3	23.4 ± 1.6	ns
Total cholesterol, mg/dL	204 ± 9	209 ± 8	202 ± 10	ns
Fasting plasma glucose, mg/dL	89 ± 8	88 ± 10	90 ± 13	ns
Creatinine, mg/dL	0.9 ± 0.1	0.9 ± 0.2	0.9 ± 0.1	ns
AST, U/L	23 ± 9	25 ± 11	22 ± 12	ns
ALT, U/L	27 ± 11	28 ± 10	27 ± 10	ns

None of these variables revealed any significant differences, indicating a well-balanced distribution among the three groups with respect to potential confounding factors. Serum concentrations of beclin-1 and mTOR levels among the three professional groups are depicted in Table 2.

**Table 2:** Serum concentrations of the autophagy biomarkers, beclin-1 and mTOR, among construction workers, airline pilots, and fitness instructors Data are expressed as mean ± standard deviation. *P<0.05 versus airline pilots; †P<0.05 versus fitness instructurs. Abbreviation: mTOR, mammalian target of rapamycin.

Biomarker	Construction workers (n = 20)	Airline pilots (n = 20)		Fitness instructors (n = 20)	P value
Beclin-1 (ng/mL)	2.4 ± 0.4*,†	1.9 ± 0.5†		3.1 ± 0.9	<0.001
mTOR (ng/mL)	5.9 ± 1.9*,†	4.4 ± 1.7†		3.5 ± 1.2	<0.001

One-way ANOVA revealed highly significant differences for both biomarkers (both P < 0.001), indicating that there are substantial variations in autophagy between the groups being compared. As per the post hoc analyses, fitness instructors exhibited the highest beclin-1 concentration, averaging at 3.1 ± 0.9 ng/mL. They were followed by construction workers with an average of 2.4 ± 0.4 ng/mL, while airline pilots registered the lowest levels at 1.9 ± 0.5 ng/mL. All of these intergroup variations were statistically significant (Tukey’s post hoc test: P < 0.05 for all pairwise comparisons). As for mTOR levels, construction workers had the highest concentration at 5.9 ± 1.9 ng/mL, followed by airline pilots at 4.4 ± 1.7 ng/mL. Fitness instructors, however, showed the lowest mTOR levels, averaging 3.5 ± 1.2 ng/mL. These differences among the groups also held statistical significance (Tukey’s post hoc test: P < 0.05 for all pairwise comparisons). We did not observe any significant associations between serum levels of autophagy biomarkers and the general characteristics of the study participants listed in Table 1 (data not shown).

## Discussion

No previous research has delved into the relationship between occupational factors and serum levels of autophagy biomarkers. As a response to this knowledge gap, we conducted a preliminary study, the findings of which brought to light two significant results. Firstly, we discovered that fitness instructors exhibited the peak activation of autophagy as demonstrated by their increased serum beclin-1 concentrations, followed closely by construction workers, while airline pilots recorded the least. Secondly, our research revealed that construction workers exhibited the highest levels of mTOR, a biomarker indicative of autophagy impairment and cellular senescence [21]. They were closely trailed by airline pilots, who showed intermediate concentrations; conversely, fitness instructors were found to have the lowest levels.

Physical exercise, which is characterized by deliberate, structured, and recurring physical activity, is recognized as a significant stimulus for autophagy [17,18]. As anticipated, our findings reveal a significantly elevated level of serum beclin-1 among fitness instructors in comparison to other professional groups. The activation of autophagy-related proteins, such as beclin-1, due to exercise has been previously documented in animals engaged in treadmill exercise [25]. In humans, research by Brandt et al. [26] indicated that both individual exercise sessions and consistent exercise training enhance the expression of autophagy markers in skeletal muscle. Moreover, regular exercise is linked with healthy aging [27], and we have previously noted an increase in serum beclin-1 levels in healthy centenarians [22]. Conversely, the role that mTOR plays in exercise seems to be multifaceted, managing both the anabolic and catabolic signaling pathways related to skeletal muscle mass [28]. This, in turn, leads to the regulation of muscle hypertrophy and muscle degradation. Noteworthy is the fact that mTOR is a component of two distinct complexes named mTORC1 and mTORC2, each having unique functions. On the one hand, mTORC1 is known to promote protein synthesis and augment cell size. On the other hand, mTORC2 plays a crucial role in the regulation of aging, cell survival, and the organization of the cytoskeleton [29]. To our knowledge, there are no previous studies investigating the correlation between human physical activity and serum mTOR levels. Our research unveiled that fitness instructors manifested the lowest serum mTOR levels, which aligns with the hypothesis that circulating mTOR in the bloodstream could potentially act as a biomarker for cellular senescence, typically resultant from compromised autophagy [21]. It is worth highlighting that exercise is recognized for mitigating the primary signs of aging [27]. Elevated mTOR levels in airline pilots and particularly in construction workers could suggest that these occupational groups are more susceptible to job-related stress, potentially fostering cellular senescence [30]. Both professions [11,31] may face stressors such as erratic work schedules, fatigue, and psychological strain, all of which might contribute to a senescent-like phenotype. Construction workers are also particularly susceptible to extreme weather conditions and airborne contaminants [31], both of which may promote senescence. This could potentially account for the elevated levels of mTOR observed within this group. Intriguingly, construction workers exhibited higher beclin-1 levels as compared to airline pilots. This phenomenon could be attributed to a heightened activation of autophagy in the former group, acting as a countermeasure to eliminate particulate matter and toxic metals [16]. Conversely, airline pilots displayed the lowest beclin-1 concentrations. Although the reasons for this observation are speculative at present, it could be due to a combination of inadequate physical activity from prolonged immobility in the cockpit and high rates of disrupted sleep, both of which have been shown to potentially impair autophagy [32].

Our study has several limitations that need to be considered when interpreting the findings. Firstly, we did not investigate the underlying mechanisms that could explain the differences in serum beclin-1 and mTOR levels among the three occupational groups. This means that we cannot establish causality or fully understand the reasons behind these observations. Unfortunately, we were unable to collect data on the levels of physical activity in the three professional groups. This omission of crucial information was primarily a result of the limitations imposed by our routine occupational medicine consultations. To tackle this issue, future research should adopt a more comprehensive approach that incorporates objective methods, such as body-worn activity monitors, to assess patterns of physical activity and sedentary behaviors. These objective approaches offer advantages over self-reported questionnaires or diary-based methods and would greatly enhance the accuracy and reliability of the data obtained. Secondly, we acknowledge the limitation of a small sample size. Therefore, it is crucial to replicate our findings with a more substantial number of participants to strengthen the validity of our conclusions. Lastly, due to budget constraints, we only analyzed two autophagy-related markers in serum samples. This may have limited our ability to capture the full picture of autophagy activity.

## Conclusions

Despite these limitations, our preliminary findings have revealed a significant variation in serum levels of autophagy biomarkers among healthy individuals, depending on their professional roles. Given the crucial role autophagy plays in maintaining health and preventing disease, it is essential to conduct further investigations. To broaden our understanding, future research should include individuals in sedentary professions, such as office workers. This approach will enable us to effectively evaluate whether autophagy biomarkers are indeed diminished within these specific professional groups, as expected. Moreover, we acknowledge the importance of conducting longitudinal studies to explore the interrelationships between occupational exposure, autophagy biomarker levels, disease-specific morbidity, and patient-reported outcomes. By undertaking such research, we can enhance our understanding of how these factors interplay and their overall impact on health in the field of occupational medicine.
